# Pro-Inflammatory Food, Gut Microbiota, and Cardiovascular and Pancreatic Diseases

**DOI:** 10.3390/biom14020210

**Published:** 2024-02-10

**Authors:** Bing Chen, Shriraj Patel, Lingyu Bao, Danial Nadeem, Chayakrit Krittanawong

**Affiliations:** 1 Department of Gastroenterology, Hepatology, and Nutrition, Geisinger Medical Center, Danville, PA 17822, USA; 2 Department of Medicine, Geisinger Medical Center, Danville, PA 17822, USA; 3 Section on Molecular Morphogenesis, Eunice Kennedy Shriver National Institute of Child Health and Human Development, National Institutes of Health, Bethesda, MD 20817, USA; 4 Cardiology Division, NYU School of Medicine and NYU Langone Health, New York, NY 10016, USA

**Keywords:** pro-inflammatory food, gut microbiota, trimethylamine-N-oxide, pancreatic diseases, cardiovascular diseases

## Abstract

Recent studies have shown that a pro-inflammatory diet and dysbiosis, especially a high level of trimethylamine-N-oxide (TMAO), are associated with various adverse health conditions. Cardiovascular diseases and pancreatic diseases are two major morbidities in the modern world. Through this narrative review, we aimed to summarize the association between a pro-inflammatory diet, gut microbiota, and cardiovascular and pancreatic diseases, along with their underlying mechanisms. Our review revealed that TMAO is associated with the development of cardiovascular diseases by promoting platelet aggregation, atherosclerotic plaque formation, and vascular inflammation. TMAO is also associated with the development of acute pancreatitis. The pro-inflammatory diet is associated with an increased risk of pancreatic cancer and cardiovascular diseases through mechanisms that include increasing TMAO levels, activating the lipopolysaccharides cascade, and the direct pro-inflammatory effect of certain nutrients. Meanwhile, an anti-inflammatory diet decreases the risk of cardiovascular diseases and pancreatic cancer.

## 1. Introduction

Emerging studies have shown that the Western dietary pattern, characterized by high saturated fats, salt, sugar, and processed food consumption, is associated with chronic metabolic inflammation [1]. The Dietary Inflammatory Index (DII) is an algorithmic scoring system that categorizes individuals’ diets based on their inflammatory potential [2]. The effect of the dietary components on inflammation was evaluated based on their impact on the level of interleukin (IL)-1β, IL-6, tumor necrosis factor (TNF)-α, C reactive protein, IL-4, and IL-10 [2]. Among thirty-seven dietary components and food products used to calculate the DII score, there are eight pro-inflammatory dietary elements, including carbohydrates, protein, total fat, saturated fatty acids, trans fat, cholesterol, iron, and vitamin B12 [2,3]. Additionally, there are twenty-nine anti-inflammatory elements, such as monounsaturated or polyunsaturated fatty acids, fiber, flavonols, and tea. Diet is considered more pro-inflammatory if the DII score is high, typically characterized by a high intake of fats, excluding vegetable oils, fruit juices, red or processed meat, sweets, sugar, and sugar-sweetened beverages in a Polish study [3]. Many studies have shown that diets with higher DII scores are associated with an increased risk of diabetes, dyslipidemia, metabolic syndrome, cardiovascular diseases (CVD), and cancers, including pancreatic cancer [4,5,6,7,8]. 

Trimethylamine-N-oxide (TMAO) is an amine oxide that is generated from choline, betaine, and carnitine, which are enriched in animal products. Gut microbiota metabolizes them to trimethylamine (TMA), which is then oxidized to TMAO in the liver [9,10]. Multiple studies have shown that TMAO level is associated with the development of many diseases, such as cancers, neurological disorders, metabolic syndrome, and CVD [11]. Food components play an important role in the human gut microbiome and TMAO. Depending on the type of diet, variations in gut microbiota and TMAO levels have been detected. Vegetarians and vegans have reduced urinary TMAO levels, which might be related to changes in the composition of the gut microbiota and lower carnitine and choline intake [12]. Further evidence shows that even when supplementing with native carnitine, the levels of TMAO in vegans remain normal, highlighting the effects of gut microbiota on TMAO levels [13].

CVD is the leading cause of illness and death worldwide. Every year, an estimated 1.5 million heart attacks and strokes occur in the United States, with 0.8 million people dying from CVD annually, accounting for one-third of all deaths [14]. In the United States, acute pancreatitis is the leading cause of hospitalization related to gastrointestinal disorders, and the incidence continues to rise, while pancreatic cancer is the third-leading cause of cancer mortality [15,16]. 

Our review aims to explore the relationship between pro-inflammatory foods, gut microbiota, TMAO, pancreatic disease, and CVD, elucidating the underlying mechanisms. 

## 2. Methods 

To identify pertinent studies, a thorough search of the PubMed/MEDLINE database was conducted, spanning from inception to July 2023. Keywords such as “pro-inflammatory food”, “anti-inflammatory food”, “pancreatic cancer”, “pancreatic steatosis”, “gut microbiota”, “trimethylamine-N-oxide”, and “cardiovascular diseases” were employed in the search. Only English-language literature was considered, encompassing a range of study types, including reviews, observational studies, case-control studies, cohort studies, clinical trials, and animal studies. Following the screening process, a narrative synthesis of the identified studies was undertaken.

## 3. Cardiovascular Diseases and TMAO 

**Figure 1:**
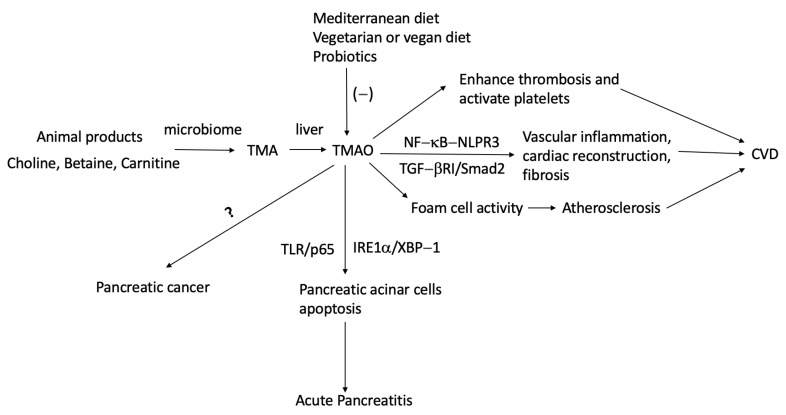
The mechanisms of the relationship between TMAO and CVD or pancreatic diseases. ? means the data are contradictory, and no conclusions can be currently made. TMA = trimethylamine, TMAO = Trimethylamine-N-oxide, NF = nuclear factor, CVD = cardiovascular disease.

TMAO has recently garnered increasing attention within the scientific community in the development of CVD. Although the exact mechanism by which TMAO influences the progression of CVD is still unclear, compelling evidence from both human and mouse model studies shows several ways it might be involved. First, TMAO was shown to directly contribute to the incidence of thrombotic events by enhancing thrombosis and activating platelets, which are crucial contributors to the pathogenesis of CVD in humans [17,18]. In line with findings from human studies, animal studies involving the transplantation of human microbiome into germ-free mice showed that high levels of TMA-converting microbes from humans caused higher levels of TMAO in recipient mice, resulting in increased platelet aggregation [19]. Second, a high TMAO level is associated with an increased overall inflammatory burden, which affects vascular inflammation and contributes to cardiac reconstruction and fibrosis [20,21,22]. Nuclear-factor (NF)-κB–NLPR3 and TGF-ßRI/Smad2 pathways are involved in this process [23,24]. Furthermore, some basic scientific studies showed that TMAO levels were correlated with atherosclerotic plaque formation due to enhanced foam cell activity [21]. Macrophage scavenger receptors linked to atherosclerosis were upregulated in mice fed with choline, TMAO, or betaine. Meanwhile, probiotics have been shown to reduce TMAO levels in different studies [25,26]. Animal studies further demonstrate that probiotics-treated rats/mice had improved cardiovascular event outcomes, such as infarct size, heart failure, and myocardial reconstruction, and lower blood pressure through decreased inflammatory biomarkers and the renin–angiotensin–aldosterone system [27,28,29] (Table 1 and Figure 1).

## 4. Pancreatic Diseases and TMAO 

In the last decade, researchers have grown aware of the importance of the gut microbiota’s relationship with pancreatitis, either through a direct pathway or indirect effects on other common causes, like hyperlipidemia. TMAO levels were reduced during acute pancreatitis in an animal study [30]. From the evidence of an in vitro study, TMAO was shown to induce apoptosis of pancreatic acinar cells, which is the hallmark of hyperlipidemia-caused early-stage acute pancreatitis, by activating oxidative stress and regulating the endoplasmic reticulum stress-associated endoribonuclease inositol-requiring enzyme 1α (IRE1α)/X-box binding protein 1 pathway [31]. Furthermore, from a hyperlipidemia acute pancreatitis mice model, TMAO was shown to enhance the injury of pancreatic cells through toll-like receptor (TLR)/p65-mediated inflammatory effects [35]. 

Unlike in pancreatitis, the role of TMAO in pancreatic cancer is still controversial. A study from human serum revealed that pancreatic cancer patients had a lower level of TMAO by using ^1^H nuclear magnetic resonance to analyze the serum metabolic profile [32]. This is further validated in the animal study. TMAO was shown to directly amplify the effector T cell activation and reduce pancreatic ductal adenocarcinoma burden by altering the tumor microenvironment in a type I interferon-dependent manner [33]. In a prospective cohort study, TMAO was associated with an odds ratio (OR) of 2.81 [95% confidence interval (CI): 1.37–5.76] for pancreatic cancer in the Shanghai cohort, while the association was not significant in the Singapore Chinese Study [34].

## 5. Pro-Inflammatory Foods and Pancreatic Diseases 

**Figure 2:**
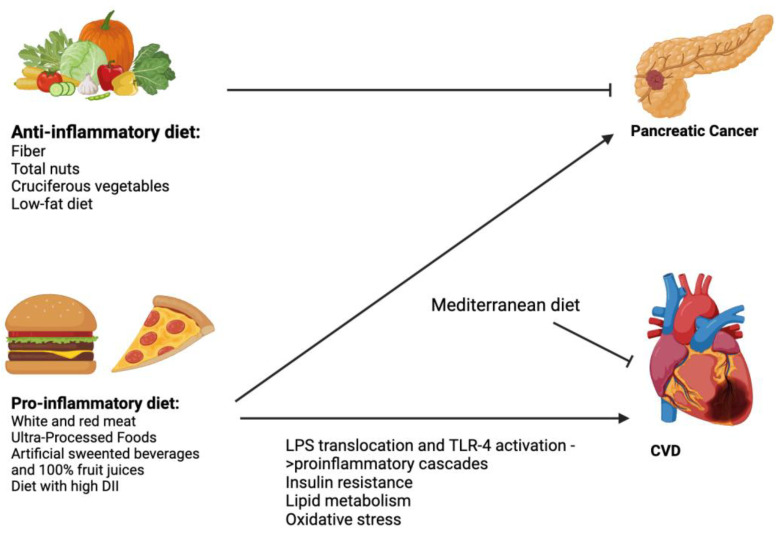
Summary of the relationship between food and pancreatic disease and CVD. Acronyms: DII = dietary inflammatory index, CVD = cardiovascular diseases, LPS: lipopolysaccharides.

A meta-analysis by Guo et al. with two prospective cohort studies and four case-control studies showed that dietary habits characterized by high inflammatory features, as indicated by a high DII, may significantly increase the risk of pancreatic cancer in individuals in the highest category with a 45% increased risk of pancreatic cancer with a relative risk (RR) of 1.45 (95% CI: 1.11–1.90) [6]. The dose-response meta-analysis revealed that for every 1-unit increase in the DII score, there was an associated increase in pancreatic cancer risk of 8% with an RR of 1.08 (95% CI: 1.002–1.166) (Table 2 and Figure 2).

Several studies have found associations between certain dietary factors and an increased risk of pancreatic cancer. A recent meta-analysis by Kim et al. analyzed twenty prospective cohort studies and showed that high consumption of white meat and red meat was associated with increased risk of pancreatic cancer [36]. The pooled RR of pancreatic cancer comparing highest versus lowest intakes of red meat was 1.09 (95% CI: 0.97–1.21); when separated by sex, a statistically significant positive association was seen for men with an RR of 1.22 (95% CI: 1.02–1.47) but not for women. In the dose-response meta-analysis, the pooled RR for 120 g per day of increased red meat intake was 1.14 (95% CI: 1.01–1.28), suggesting a borderline significant association between red meat intake and cancer. The pooled RR of pancreatic cancer comparing highest vs. lowest intake of white meat was 1.14 (95% CI: 1.03–1.27). Furthermore, high intake of ultra-processed foods was associated with increased risk of various cancers, including pancreatic cancer, with a hazard ratio (HR) of 1.49 (95% CI: 1.07–2.07) in a prospective cohort study by Zhong et al. [37]. Pan et al. found a linear dose-response association between consumption of artificially sweetened beverages and 100% fruit juices and pancreatic cancer [42]. 

On the other hand, certain foods have not been found to be associated with increased risk of pancreatic cancer. For example, in Arafa et al.’s meta-analysis, which included data from the Japan Collaborative Cohort Study and four other prospective cohort studies, the consumption of milk, cheese, and yogurt was not found to be associated with the risk of pancreatic cancer with HRs of 0.95 (95% CI: 0.82–1.11), 1.16 (95% CI: 0.87–1.55), and 0.91 (95% CI: 0.79–1.05), respectively [39].

Similarly, as shown by Zhang et al. in a meta-analysis including 12 case-control and two cohort studies, total protein intake had no significant association with the risk of pancreatic cancer with an RR of 1.02 (95% CI: 0.85–1.22); however, in a subgroup analysis by protein type, although not statistically significant, the opposite association was found with animal protein intake with an RR of 1.37 (95% CI: 0.93–2.01) and vegetable protein intake with an RR of 0.78 (95% CI: 0.54–1.14) [40]. A meta-analysis by Gao et al. examined 25 studies and showed that there was no appreciable link between fish intake and pancreatic cancer risk with an RR of 1.00 (95% CI: 0.93–1.07) [41]. A meta-analysis by Bae et al. with twelve cohort studies and 10,587 pancreatic cancer incidents showed no increased risk of pancreatic cancer among patients with the highest versus the lowest level of coffee consumption with a summary RR of 0.98 (95% CI: 0.88–1.10) [38].

Finally, some foods have been shown to have a protective effect. Jiao et al. had a randomized controlled trial with 48,835 postmenopausal women aged 50 to 70 years in the United States between 1993 and 1998, showing that a low-fat dietary intervention was associated with a reduced risk of pancreatic cancer in patients with a baseline body mass index of 25 kg/m^2^ or higher with an HR of 0.71 (95% CI: 0.53–0.96) [43]. Li et al. performed a meta-analysis showing that high intake of cruciferous vegetables is associated with a significantly decreased risk of pancreatic cancer with an OR of 0.78 (95% CI: 0.64–0.91) [44]. Nucci et al. performed a meta-analysis that showed that higher dietary fiber intake is associated with a significantly lower risk of pancreatic cancer in both fixed and random effects models. In the fixed effect model, the pooled effect size was 0.75 (95% CI: 0.69–0.82); in the random effect model, the pooled effect size was 0.63 (95% CI: 0.53–0.76) [45]. Relatedly, Naghshi et al. analyzed 42 articles on cancer risk and 9 articles on cancer mortality; based on a dose-response analysis, a 5 g/d increase in total nut intake was associated with a 6% lower risk of pancreatic cancer with a pooled effect size of 0.94 (95% CI: 0.89–0.99) [46]. Fu et al. conducted a meta-analysis of 16 studies showing a significant association between folate intake and decreased risk of pancreatic cancer, with a pooled OR of 0.82 (95% CI: 0.69–0.97) [47]. However, the association was observed only in case-control studies [OR = 0.78, (95% CI: 0.65–0.93)], but not in cohort studies [RR: 0.85, (95% CI: 0.66–1.09)]. A dose-response meta-analysis showed that an incremental intake of folate (100 μg/day) was marginally associated with the risk of pancreatic cancer, with a pooled OR of 0.97 (95% CI: 0.93–1.00).

In the exploration of the influence of specific dietary components on pancreatic diseases, it is essential to delve into the role of leptin—a prominent adipokine linked to obesity that regulates both food intake and energy metabolism [51]. Leptin is involved in the storage of triglycerides within adipocytes in the pancreas, contributing to the development of pancreatic steatosis [52,53]. Furthermore, elevated levels of leptin have been identified in patients with pancreatic cancer, playing a potential role in cancer progression [54]. Importantly, pancreatic steatosis demonstrates a significant association with diabetes mellitus and hepatic steatosis. Leptin emerges as a mediator in establishing connections between insulin resistance, hepatic steatosis, and pancreatic steatosis [51,55].

## 6. Inflammatory Foods and Cardiovascular Diseases

The association between food and CVD has been more extensively investigated. However, in comparison to the examination of diet and pancreatic diseases, there has been a shift towards studying dietary patterns rather than specific food items, such as red meats and vegetables, in relation to CVD and mortality. This transition recognizes that the impact of diet on health is a complex interplay of various factors, and focusing solely on individual food items may not adequately capture this complexity [56]. 

Particularly long-standing has been the support for the dietary pattern of a Mediterranean diet. This dates back to a groundbreaking clinical trial in Lyon, France, between 1988–1992 by de Lorgeril et al., known as the Lyon Diet Heart Study, which showed that a Mediterranean-style diet led to a remarkable 72% reduction in the risk of recurrent heart attacks and substantial reduction in overall mortality compared to the control group [48]. This was followed by the Primary Prevention of CVD with Mediterranean Diet Supplemented with Extra-Virgin Olive Oil or Nuts Trial by Estruch et al., a multicenter trial where patients aged 55 to 80 years at high cardiovascular risk but no existing CVD were assigned to one of three diets: Mediterranean diet with extra-virgin olive oil, Mediterranean diet supplemented with nuts, or the control diet with low-fat items [49]. The primary outcome measured was the occurrence of major cardiovascular events, including myocardial infarction, stroke, or death from cardiovascular causes. After a median follow-up of 4.8 years, the trial was stopped based on a pre-planned interim analysis. The results showed that the occurrence of primary endpoints was lower in the groups assigned to the Mediterranean diet supplemented with extra-virgin olive oil (3.8%) and Mediterranean diet supplemented with nuts (3.4%), compared to the control group (4.4%). In the intention-to-treat analysis, the hazard ratio for major cardiovascular events was 0.69% (95% CI: 0.53–0.91) for the Mediterranean diet with extra-virgin olive oil and 0.72 (95% CI: 0.54–0.95) for the Mediterranean diet with nuts, compared to the control diet. The ketogenic diet, including variations like the Atkins and Dukan diets, characterized by restricting carbohydrate intake, has gained attention for its potential effects on weight loss and the management of diabetes mellitus [57]. An umbrella review of 17 meta-analyses comprising 68 randomized controlled trials showed that the ketogenic diet is associated with improvements in several cardiometabolic parameters, including reduced triglycerides, decreased body weight, and hemoglobin A1C levels [58]. However, this study showed that the ketogenic diet is associated with low-density lipoprotein cholesterol. Clinical trials are needed to study the effect of the ketogenic diet on cardiovascular outcomes. 

In a cross-sectional study by Namazi et al., a pro-inflammatory diet (as determined by the DII) was examined in patients with Type 2 Diabetes Mellitus (T2DM) compared to non-T2DM cases [4]. Patients were divided into four quartiles based on DII scores as calculated using a food frequency questionnaire; results showed that higher DII scores were associated with a 61% increased risk of T2DM after adjusting for confounding factors. DII was also significantly associated with obesity and dyslipidemia in both diabetic and non-diabetic cases. However, no significant association was found between DII and metabolic syndrome or hypertension in either group. The association between DII and CVDs was only significant in diabetic patients, suggesting that a pro-inflammatory diet increases the risk of T2DM and certain cardiometabolic risk factors, which are more pronounced among patients with T2DM compared to non-diabetics. A cross-sectional study by Aslani et al. examined 10 prospective cohort studies in a systematic review, showing that individuals with the highest DII score category had a 29% increased risk of cardiometabolic disease mortality compared to those with the lowest DII category with an HR of 1.29 (95% CI: 1.18–1.41). A significant association was also found between the DII score and metabolic syndrome with an OR of 1.13 (95% CI: 1.03–1.25) [5]. Shah et al. performed a standardized case-control study investigating the effects of a vegan diet compared to an American Heart Association-recommended diet on high-sensitivity C-reactive protein levels in patients with coronary artery disease [50]. One hundred participants were randomized to either diet for 8 weeks and results from a linear regression model showed that the vegan diet led to 32% lower high-sensitivity C-reactive protein (*p* = 0.02). 

Moreover, other studies have analyzed the potential contributions of medicinal plants in mitigating the absorption of high-fat foods. In a comprehensive review by de Freitas Jr. and de Almeida Jr., 23 articles were reported that identified an anti-obesity effect through in vivo and/or in vitro biological tests [59]. These studies elucidated the impact of plant metabolites on the delayed absorption of dietary fat. Of particular interest, *Achyranthes aspera* L. seeds were found to delay the intestinal absorption of fat in the diet by inhibiting pancreatic amylase and lipase activity [60]. *Moringa oleifera* leaves demonstrated a significant reduction in the atherogenic index, effectively countering the hyperlipidemic effects induced by a high-fat diet in obese rats [61]. Furthermore, fruits, containing a rich source of compounds referred to as flavonoids—polyphenol compounds characterized by two aromatic rings—have been postulated to reduce cardiovascular mortality by decreasing cholesterol transport, high-density lipoprotein metabolism, and high-density lipoprotein function while also exerting antioxidant effects and modifying gut microbiota [62,63,64,65]. Li et al. detail how specific flavonols, a subtype of flavonoids featuring an added ketone group and naturally occurring in fruits, have inhibitory effects on critical cellular pathways, including PI3-Kinase [66]. These pathways play a pivotal role in the mechanisms associated with chronic inflammation and, consequently, pancreatic and CVD. However, as emphasized by Li et al., further clinical trials specifically focusing on the medicinal potential of flavonoids remain a promising field to be explored. Presently, ongoing research is predominantly directed towards elucidating their anti-inflammatory mechanisms, which are undoubtedly crucial to understanding their full therapeutic potential. 

## 7. Mechanism of Pro-Inflammatory Food 

Given the above discussions, it is well-established that pro-inflammatory diets lead to a statistically significant increase in risk for both pancreatic and CVD, as objectively investigated using the DII. As such, Tsoupras et al. posited that “Inflammation, not Cholesterol, Is a Cause of Chronic Disease” [67]. However, the pathogenesis of these conditions involves intricate interactions between the gut microbiota and the host’s physiological processes.

One principal pathway involves lipopolysaccharides, confined to the gut by tight junctions between epithelial cells and mucosal immunity [68]. Altered gut permeability allows macrophages to infiltrate and produce inflammatory cytokines that cause a local inflammatory response, allowing the translocation of lipopolysaccharides into systemic circulation—a phenomenon referred to as “leaky gut” [69]. Once in the bloodstream, lipopolysaccharides bind toll-like receptor 4 on immune cells, a pattern recognition receptor, leading to up-regulation of NF-κB and thus an inflammatory response [70]. The activation of TLR-4 triggers pro-inflammatory cascades in both local and distant tissues, leading to systemic inflammation [71]. 

Some macronutrients, such as refined carbohydrates and trans-fat, are directly associated with an increase in circulating levels of pro-inflammatory cytokines and increased oxidative stress [72,73]. The ingestion of these macronutrients is linked to the elevated activity of pro-inflammatory transcriptional factors, such as NF-κB, activating protein-1, and early growth response protein 1, which are associated with an increase in reactive oxygen species generation [73,74]. This is accompanied by elevated circulating levels of inflammatory cytokines, including IL-18, IL-6, and TNF-α [75,76,77].

Short-chain fatty acids, particularly acetate, derived from complex carbohydrates, impact energy regulation, lipid metabolism, glucose homeostasis, gut inflammation, and blood pressure [78,79,80,81]. Bile acids modulate lipid and glucose/insulin metabolism and inflammation [82]. The gut microbiota further modifies primary bile acids to produce secondary bile acids, which interact with various receptors to influence cardiometabolic phenotypes and disease susceptibility [83,84,85,86]. Notably, alterations in levels of bile acids in plasma have been correlated with insulin resistance in type 2 diabetes mellitus [82]. 

Chronic inflammation influences the onset, development, and progression of various diseases by driving endothelial dysfunction, triggering pro-inflammatory cytokine production, and increasing oxidative stress [87]. According to Rotariu et al., heightened oxidative stress alters molecular pathways in a way that contributes to the pathophysiology of CVD [88]. Reactive oxygen species contribute to the altered structure and function of hypertrophic myocytes [89]. Additionally, reactive oxygen species are linked to angiotensin-II-mediated cardiac hypertrophy, NADPH-oxidase activation, and increased collagen synthesis, leading to cardiac fibrosis. Furthermore, reactive oxygen species formation triggers endothelial inflammation, foam cell formation, and subsequent atherosclerotic plaque formation [90]. As highlighted by Rotariu et al., oxidative modification of low-density lipoprotein cholesterol is recognized as a marker of oxidative stress [88]. According to Nigam et al., metabolic disorders, neurodegenerative disease, and cancer are also intricately linked to chronic inflammation [91]. Within the context of cancer, chronic inflammation plays a distinct role in the proliferation of diverse cancer types, including breast cancer, lung cancer, liver cancer, colorectal cancer, and pancreatic cancer.

## 8. Conclusions

In summary, it is evident that a pro-inflammatory diet can elevate the risk of both pancreatic cancer and CVD, whereas adopting an anti-inflammatory dietary approach is linked to a reduced likelihood of these diseases. The gut microbiota, particularly the elevated levels of TMAO, emerges as a significant player in the development of CVD and may also be associated with acute pancreatitis. Encouragingly, an anti-inflammatory diet is associated with lower TMAO levels. Other potential mechanisms through which a pro-inflammatory diet exerts its effects include the initiation of inflammatory responses triggered by lipopolysaccharides and the direct inflammatory effect of certain micronutrients.

## Figures and Tables

**Table 1:** Summary of the key studies in the relationship between TMAO and CVD or pancreatic diseases.

First Author	Type of Study	Patients, n	Outcomes
Skye et al. [19]	Animal and human study	N/A	TMA/TMAO-enhanced platelet reactivity and thrombosis potential.
Haghikia et al. [20]	Cohort study	593	High TMAO is associated with increased risk of adverse cardiovascular events in patients with recent first-ever stroke with an adjusted HR of 3.3 (95% CI: 1.2–10.9) during 1-year follow-up.
Chen et al. [23]	Cell and animal study	N/A	TMAO promoted vascular inflammation by activating the NLRP3 pathway.
Yang et al. [24]	Animal study	N/A	TMAO accelerated cardiac fibrosis by activating the TGF-βRI/Smad2 pathway.
Koeth et al. [13]	Animal and human cohort study	2595	Chronic dietary L-carnitine supplementation in mice enhanced the synthesis of TMAO and increased atherosclerosis. In humans, plasma L-carnitine levels predicted risks for prevalent CVD and incident major adverse cardiovascular events only in subjects with high TMAO levels.
Li et al. [30]	Animal study	N/A	TMAO level was decreased in rats with experimental acute pancreatitis.
Yang et al. [31]	Animal study	N/A	TMAO increased the incidence of acute pancreatitis by activating the inositol-requiring enzyme 1α (IRE1α)/X-box binding protein 1 pathway.
OuYang et al. [32]	Human study	17 with pancreatic cancer vs. 24 controls	Pancreatic cancer patients had lower plasma levels of TMAO.
Mirji et al. [33]	Animal and human study	N/A	The abundance of TMAO-generating bacteria correlated with improved survival and response to anti-programmed cell death protein 1 in a mouse model of pancreatic cancer.
Huang et al. [34]	Human prospective cohort study	129 cases and 258 controls in the Shanghai Cohort, 58 cases and 104 controls in Singapore Chinese Health Study	TMAO is associated with an OR of 2.81 (95% CI: 1.37–5.76) for pancreatic cancer in the Shanghai cohort, while the association was not significant in the Singapore Chinese Study.
Filippis et al. [12]	Human study	153	Vegetarian and vegan diet was associated with lower levels of TMAO.
Qiu et al. [25]	Animal study	N/A	The use of probiotics reduced the serum TMAO levels.
Tenore et al. [26]	Randomized controlled trial	90	Lactofermented Annurca apple puree intake reduced plasma TMAO levels.
Costanza et al. [27]	Randomized controlled trial	N/A	Among heart failure patients, treatment with probiotics was associated with a reduction in total cholesterol levels, uric acid levels, and left atrial diameter, and an improvement in left ventricular ejection fraction.
Khalesi et al. [28]	Meta-analysis	543	Probiotic therapy led to a significant reduction of systolic blood pressure by 3.56 mmHg and diastolic blood pressure by 2.38 mmHg.

Acronyms: TMA = trimethylamine, TMAO = Trimethylamine-N-oxide, NF = nuclear factor, CVD = cardiovascular disease, OR = odds ratio, CI = confidence interval.

**Table 2:** Key studies of inflammatory food and pancreatic diseases and CVD.

First Author	Types of Study	Patients, n	Outcomes
Guo et al. [6]	Meta-analysis with 2 prospective cohort studies and 4 case-control studies	634,705	Highest DII category was associated with 45% increased risk of pancreatic cancer (PC) with an RR of 1.45 (95% CI: 1.11–1.90). Every 1-unit increase in the DII score increased PC risk by 8% with an RR of 1.08 (95% CI: 1.002–1.166).
Kim et al. [36]	Meta-analysis	3,934,909	The pooled RR for pancreatic cancer in highest vs. lowest intakes of red meat and white meat was 1.09 (95% CI: 0.97–1.21) and 1.14 (95% CI: 1.03–1.27), respectively.
Zhong et al. [37]	Prospective cohort study	98,265	High intake of ultra-processed foods was associated with PC with an HR of 1.49 (95% CI:1.07–2.07).
Bae et al. [38]	Meta-analysis (12 cohort studies)	3,230,053 with 10,587 pancreatic cancer incidents	Summary RR of PC risk for the highest vs. the lowest level of coffee consumption was 0.98 (95% CI: 0.88–1.10).
Arafa et al. [39]	Meta-analysis with 5 prospective studies	N/A	Milk, cheese, and yogurt were not associated with reduced risk of PC with an HR of 0.95 (95% CI: 0.82–1.11), 1.16 (95% CI: 0.87–1.55), 0.91 (95% CI: 0.79–1.05), respectively.
Zhang et al. [40]	Meta-analysis (12 case-control and 2 cohort studies)	77,156	Total protein intake had no significant association with the risk of pancreatic cancer with an RR = 1.02 (95% CI: 0.85–1.22). Although not statistically significant, the opposite association was found in animal protein intake (RR:1.37, 95% CI: 0.93–2.01) and vegetable protein intake (RR:0.78, 95% CI: 0.54–1.14).
Gao et al. [41]	Meta-analysis of 25 studies	1,258,913	No appreciable link between fish intake and PC risk (RR: 1.00, 95% CI: 0.93–1.07).
Pan et al. [42]	Systematic review and 8 prospective cohort studies	1,594,301	There was a linear dose-response association between artificially sweetened beverages and 100% fruit juices and the risk of pancreatic cancer.
Jiao et al. [43]	Randomized controlled trial	48,835	Low-fat dietary intervention was associated with a reduced risk of pancreatic cancer in patients with body mass index ≥ 25 kg/m^2^ with an HR of 0.71 (95% CI: 0.53–0.96).
Li et al. [44]	Meta-analysis with five case-control studies	3207	High intake of cruciferous vegetables is associated with significantly decreased risk of pancreatic cancer (OR: 0.78, 95% CI: 0.64–0.91)
Nucci et al. [45]	Meta-analysis	343,120	Higher dietary fiber intake is associated with a significantly lower risk of pancreatic cancer with a pooled effect size of 0.63 (95% CI: 0.53–0.76).
Naghshi et al. [46]	Meta-analysis with 51 articles	1,739,414	A 5-g/d increase in total nut intake was associated with a 6% lower risk of pancreatic cancer
Fu et al. [47]	Meta-analysis of 16 studies	1,009,374	There was a significant association between folate intake and decreased risk of PC, with a pooled odds ratio of 0.82 (95% CI: 0.69–0.97). However, the association was observed only in case-control studies (OR: 0.78, 95% CI: 0.65–0.93), but not in cohort studies (RR: 0.85, 95% CI: 0.66–1.09).
Lorgeril et al. [48]	Randomized controlled trial	605	The Mediterranean diet was associated with reduced all-cause and cardiovascular (*p* = 0.01) mortality and the combination of recurrent myocardial infarction and cardiac death (*p* < 0.0001).
Estruch et al. [49]	Multicenter randomized controlled trial	7447	The hazard ratio for major cardiovascular events was 0.69% (95% CI: 0.53–0.91) for the Mediterranean diet with extra-virgin olive oil and 0.72 (95% CI: 0.54–0.95) for the Mediterranean diet with nuts, compared to the control diet.
Namazi et al. [4]	Cross-sectional analysis	9039	Results showed that higher DII scores were associated with a 61% (95% CI: 1.27–2.05) increased risk of T2DM after adjusting for confounding factors.
Aslani et al. [5]	Systematic review	291,968	Individuals with the highest DII score category had a 29% increased risk (HR: 1.29, 95% CI: 1.18–1.41) of cardiovascular and metabolic disease mortality compared to those with the lowest DII category.
Shah et al. [50]	Prospective cohort study	100	A vegan diet led to 32% lower high-sensitivity C-reactive protein (*p* = 0.02) compared to the American Heart Association-recommended diet in patients with coronary artery disease.

Acronyms: RR = Relative Risk, HR = hazard ratio, OR = odds ratio, Pancreatic cancer = PC, DII = dietary inflammatory index.
